# Alleviating Effects of *Zophobas morio* and *Tenebrio molitor* Larvae Protein Powder on Dextran Sodium Sulfate-Induced Inflammatory Bowel Disease in Mice

**DOI:** 10.3390/ijms27031405

**Published:** 2026-01-30

**Authors:** Ziqi Yang, Xianhui Yang, Juan Du, Shangwei Li, Jia Yu, Fei Qiao, Feng Zhu, Bangyan Song, Haiyan Zhang, Heng Luo, Ying Cao

**Affiliations:** 1 Medical College, Guizhou University, Guiyang 550025, China; 18005488169@163.com (Z.Y.); 13540024581@163.com (X.Y.); 2 Institute of Entomology, Guizhou University, Guiyang 550025, China; juandudj@163.com (J.D.); swlii@163.com (S.L.); 3 Natural Products Research Center of Guizhou Province, Guiyang 550014, China; yu_jia@gmc.edu.cn (J.Y.); luo_heng@gmc.edu.cn (H.L.); 4 Guizhou Key Laboratory of Agricultural Biosecurity, Institute of Plant Protection, Guizhou Academy of Agricultural Sciences, Guiyang 550006, China; 11016078@zju.edu.cn (F.Q.); gzzbszf@163.com (F.Z.); bangyansong2024@163.com (B.S.); haiyanz94@163.com (H.Z.)

**Keywords:** inflammatory bowel disease, *Zophobas morio* Larvae, *Tenebrio molitor* Larvae, alleviating effect

## Abstract

Inflammatory Bowel Disease (IBD) is a chronic, recurrent intestinal inflammatory disorder with an unclear etiology. Current pharmaceutical therapies for IBD still have several side effects, necessitating safer and more effective strategies. Edible insects are novel nutritional and bioactive resources with potential anti-inflammatory properties, but the effects of *Zophobas morio* larvae (ZML) and *Tenebrio molitor* larvae (TML) on IBD remain insufficiently explored. A 3% dextran sodium sulfate (DSS)-induced mouse colitis model was established to evaluate ZML protein powder (ZMLpp) and TML protein powder (TMLpp). Disease Activity Index (DAI), colon length, spleen weight, histopathology, inflammatory cell infiltration (LCA/MPO staining), inflammatory cytokines (*Ccl2*, *Cxcl1*, *Ptgs2*, *Nf-κb*), and intestinal microbiota (16S rRNA sequencing) were determined. The result showed that compared with the DSS group, both ZMLpp and TMLpp significantly reduced DAI, mitigated weight loss and hematochezia/diarrhea, restored colon length, attenuated mucosal damage, and preserved goblet cells and crypts, inflammatory cell infiltration, downregulated cytokine expression, improved fecal microbiota dysbiosis, such as increased abundance of beneficial bacteria like *Akkermansia*. These findings demonstrate that both ZMLpp and TMLpp alleviate DSS-induced colitis by inhibiting inflammation and modulating the microbiota, supporting their application in IBD therapy and the development of anti-colitis functional foods or pharmaceuticals.

## 1. Introduction

Inflammatory bowel disease (IBD) is a chronic, recurrent disease characterized by persistent, widespread inflammation of the intestinal mucosal lining and submucosa, resulting in symptoms such as abdominal pain, chronic diarrhea, rectal bleeding, and weight loss [1]. Although previous reports have indicated that the disease pathology is caused by a combination of environmental factors, genetic specificity, and intestinal flora imbalance, the exact etiology remains complex and uncertain [2,3], Recent studies have also shown that it is related to the following factors: early-life influences; diet, food, and nutritional exposures; urbanization and air pollution; smoking; appendectomy; medications; psychological stress; sleep; and latitude and geography [4]. In the past decade, IBD has become increasingly prevalent worldwide, and the incidence continues to rise, leading to an exponential impact on healthcare systems. Research on the diagnosis and treatment of IBD has gradually become a hot topic. In 2025, studies related to IBD diagnostic markers will include galectin-3 [5], inflammatory markers [6], fecal gut microbiota, and amino acids [7]. Emerging therapeutic strategies, ranging from immune modulation and intestinal barrier restoration to microbiota intervention, metabolic pathway targeting, and ROS scavenging, have been developed to address core pathogenic mechanisms of IBD, thereby establishing a multi-dimensional, precision-oriented therapeutic landscape [8]. Additionally, newer artificial intelligence (AI)-based technologies are currently being developed and are likely to play an important role in future diagnosis and management of IBD [9]. But Pharmaceutical treatments of IBD mainly depend on the 5-aminosalicylate agents, corticosteroids, nonsteroidal anti-inflammatory drugs, and immunosuppressive agents, which may lead to distinct side effects and higher recurrence rates [10], including headache, anorexia, nausea, vomiting, and non-bloody diarrhea [11]. Therefore, the development of safer and more effective therapeutic strategies for IBD has become an urgent clinical need to be addressed.

It is estimated that more than 2000 insect species are consumed by humans, primarily in tropical countries [12]. Currently, edible insects have been identified as potential future foods due to their high levels of vitamin B_12_, iron, zinc, fiber, essential amino acids, omega-3 and omega-6 fatty acids, and antioxidants [13]. They can be consumed in their natural form or as an additive. In recent years, insects have not only been used as a food resource but are also known to have other beneficial effects on human health, potentially supplying compounds for medication [14]. The drug activities detected include potent antimicrobials against antibiotic-resistant bacteria and HIV, as well as anticancer, antiangiogenic, anticoagulant, and wound-healing activities [15]. Among the various insect species, *Zophobas morio* larvae (ZML, also known as super mealworms) and *Tenebrio molitor* larvae (TML, also known as yellow mealworms) have been predominantly studied as a source of protein and fat for different animal species due to their qualitative and quantitative amino-acid and fatty-acid profiles [16,17]. In addition, the safety of these larvae as a raw material for food production has been determined. The European Food Safety Authority (EFSA) validated *Tenebrio molitor* as an edible insect in 2021 [18]. Presently, insects are not only regarded as a nutritional carrier but also considered to have infinite potential as a raw material for medicines. It is reported that *Zophobas morio* exhibits antioxidant capacity [19] and anti-inflammatory activity [20]. However, to date, no studies have confirmed the anti-inflammatory effect of *Zophobas morio* in colitis. It has also been reported that TML plays crucial roles in combating Alzheimer’s disease [21], obesity [22], and cancer [23], and it demonstrates antioxidant and anti-inflammatory properties [24]. Recent individual studies also showed that both TML powder and TML oil effectively alleviate colitis symptoms in mice induced by dextran sodium sulfate (DSS) [25,26]. In this present study, ZML protein powder (ZMLpp) and TML protein powder (TMLpp) were orally administered to a mouse colitis model induced by DSS. This study aimed to investigate the efficacy of ZML and TML in alleviating IBD symptoms by comparing gross pathological and histopathological findings and the expression levels of inflammatory cytokines in mouse tissues, and to clarify the improvement effects of ZML and TML on intestinal microflora.

## 2. Results

### 2.1. ZMLpp and TMLpp Effectively Alleviated Disease Activity Index (DAI) in DSS-Induced IBD Mice

**Figure 1:**
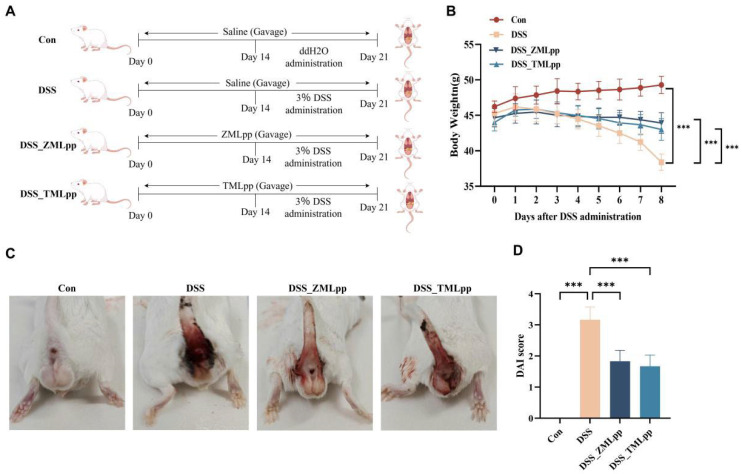
ZMLpp and TMLpp effectively alleviated DAI in DSS-induced IBD mice. (**A**) Experimental scheme illustrating the administration of ZMLpp and TMLpp in this study. (**B**) Body weight of mice in each group after DSS administration. (**C**) Characteristic images of hematochezia of mice in each group. (**D**) DAI score of mice in each group at the end of the experiment. *n* = 8 per group. Data are presented as mean ± SD. *** *p* < 0.001. Con: Control; DSS: dextran sodium sulfate; ZMLpp: *Zophobas morio* larvae protein powder; TMLpp: *Tenebrio molitor* larvae protein powder; DAI: disease activity index; IBD: Inflammatory Bowel Disease.

The IBD mouse model was established using 3% DSS, and ZMLpp and TMLpp supplements were administered via gavage to evaluate their impacts on colitis (Figure 1A). Following DSS administration, the disease activity index (DAI) score was checked daily, along with body weight, diarrhea, and hematochezia, to assess the extent of intestinal damage. Mice in the DSS, DSS_ZMLpp, and DSS_TMLpp groups began to have loose stools three days after DSS administration and subsequently showed signs of bloody stools. On the 7th day after DSS treatment, all DSS-treated groups presented symptoms of bloody stools, diarrhea, and weight loss (Figure 1B,C). At the end of the experiment, DAI scores in the DSS group were significantly higher than those in the control group. However, in the DSS_ZMLpp and DSS_TMLpp groups, this increase was attenuated, which suggests that ZMLpp or TMLpp supplementation reduced DAI scores in colitis mice (Figure 1D). The above results indicate that ZMLpp and TMLpp effectively alleviated the symptoms of colitis induced by DSS.

### 2.2. ZMLpp and TMLpp Improved the Shortening of the Colon Length and Reduced the Severity of Colitis in DSS-Induced Colitis Mice

**Figure 2:**
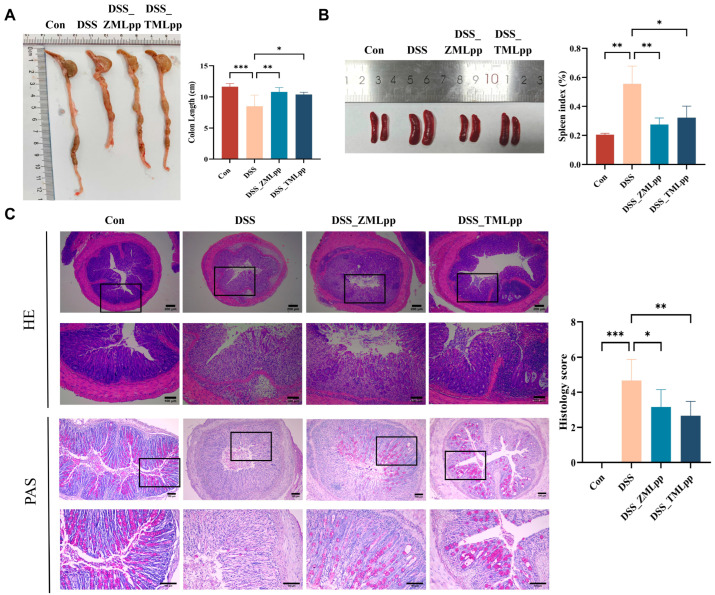
ZMLpp and TMLpp shortened colonic length and reduced the severity of DSS-induced colitis in mice. (**A**) Macroscopic morphology of the colon and colon length of mice in each group. (**B**) Macroscopic morphology of spleen and spleen index of mice in each group. (**C**) Representative images of H&E and PAS staining of mouse colon tissues and associated histological scores in each group. *n* = 8 per group. Data are presented as mean ± SD. * *p* < 0.05, ** *p* < 0.01, *** *p* < 0.001. Con: Control; DSS: dextran sodium sulfate; ZMLpp: *Zophobas morio* larvae protein powder; TMLpp: *Tenebrio molitor* larvae protein powder; H&E: Hematoxylin and eosin; PAS: Periodic acid-Schiff.

The colon lengths of the DSS group were significantly shorter than those of the control group. However, the colon lengths in the DSS_ZMLpp and DSS_TMLpp groups were significantly longer than those in the DSS group (Figure 2A). Spleen weights were significantly higher in the DSS group compared to the control group, while the spleen weights in the DSS_ZMLpp and DSS_TMLpp groups were significantly lower (Figure 2B). Hematoxylin and eosin (H&E) and Periodic acid-Schiff (PAS) staining revealed severe colitis in the DSS group. It was manifested by shortened and lost crypts, disruption of the crypt structure, a reduction in epithelial cells and goblet cells, and increased infiltration of inflammatory cells compared to the control group. Relative to the DSS group, ZMLpp and TMLpp treatment significantly reduced the severity of colitis and countered the decrease in goblet cell number caused by DSS. Histopathological scores in mice of the DSS_ZMLpp or DSS_TMLpp groups were significantly lower than those in mice treated with DSS alone (Figure 2C). This indicated that both ZMLpp and TMLpp could effectively repair the pathological damage of colonic tissue induced by DSS in mice and alleviate intestinal inflammation.

### 2.3. ZMLpp and TMLpp Reduced Inflammatory Cell Infiltration and Suppressed the Levels of Inflammatory Cytokines in the Colon of DSS-Induced Colitis Mice

**Figure 3:**
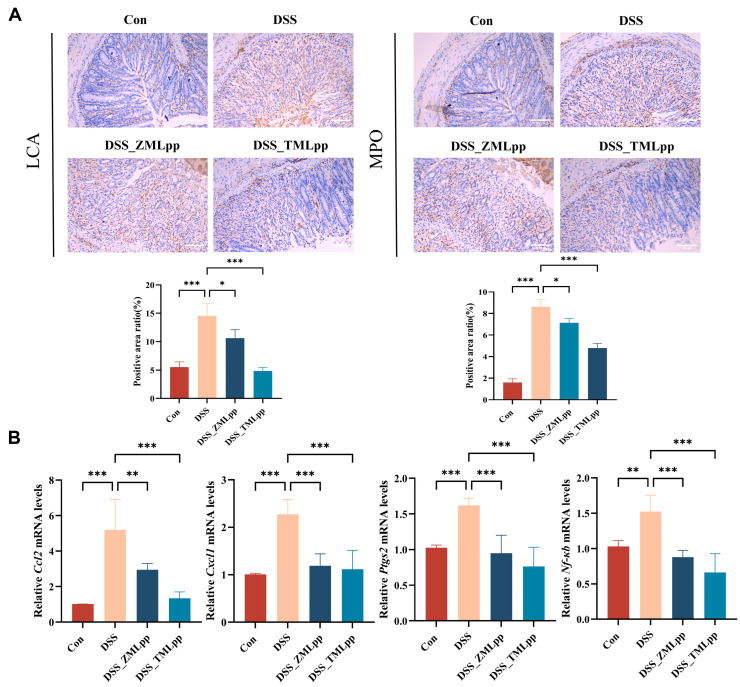
ZMLpp and TMLpp reduced inflammatory cell infiltration and suppressed the levels of inflammatory cytokines in the colon of DSS-induced colitis mice. (**A**) Representative images of LCA and MPO IHC staining and the number of LCA- and MPO-positive expression cells in the mouse colon tissue of each group. (**B**) Relative mRNA expression levels of *Ccl2*, *Cxcl1*, *Ptgs2*, and *Nf-κb* in the mouse colon tissue of each group were detected by RT-qPCR. *n* = 8 per group. Data are presented as mean ± SD. * *p* < 0.05, ** *p* < 0.01, *** *p* < 0.001. Con: Control; DSS: dextran sodium sulfate; ZMLpp: *Zophobas morio* larvae protein powder; TMLpp: *Tenebrio molitor* larvae protein powder; LCA: Leukocyte Common Antigen; MPO: Myeloperoxidase.

Leukocyte Common Antigen (LCA) and Myeloperoxidase (MPO) immunohistochemical staining were employed to detect the number of infiltrating leukocytes and neutrophils in the colonic tissues of mice in each group. Compared with the control group, the number of LCA- and MPO-positive cells in the lamina propria of the colonic mucosa was significantly increased in the DSS group, indicating a large number of leukocytes and neutrophils infiltrating the colonic tissues of DSS-induced colitis mice. Notably, after intervention with ZMLpp and TMLpp, the number of LCA- and MPO-positive cells in the colonic tissues of mice in the DSS_ZMLpp and DSS_TMLpp groups was reduced (Figure 3A). This suggests that ZMLpp and TMLpp treatment can effectively inhibit DSS-induced inflammatory cell infiltration and exert a modest improvement in IBD. To further explore the anti-inflammatory effects of ZMLpp and TMLpp, we also measured the relative mRNA levels of inflammatory cytokines in the colon. As shown in Figure 3B, both ZMLpp and TMLpp significantly reduced the mRNA expression levels of *Ccl2*, *Cxcl1*, *Ptgs2, and Nf -κb* compared to the DSS group.

### 2.4. The Regulatory Effect of ZMLpp and TMLpp on Fecal Microbial Structure

**Figure 4:**
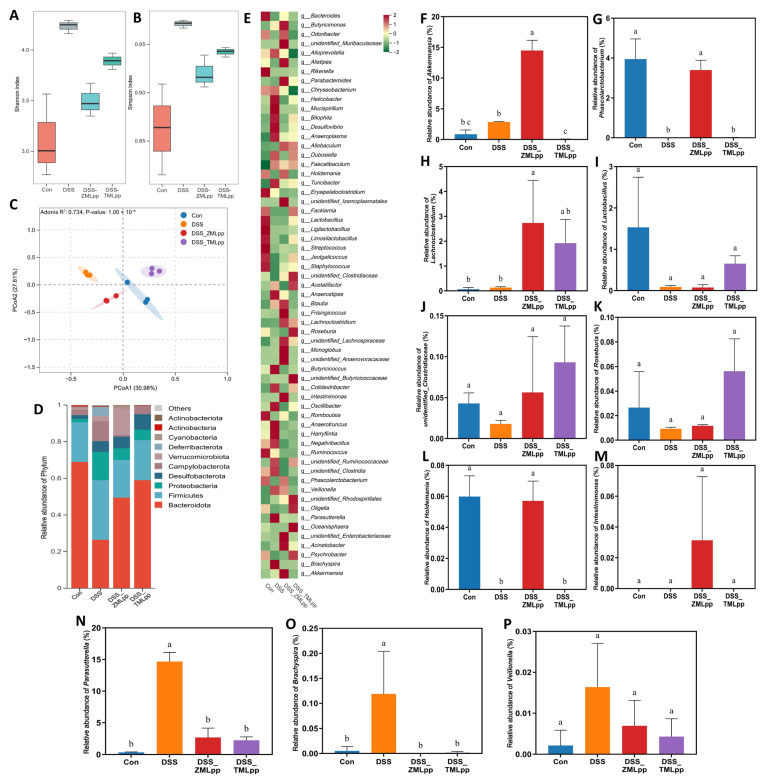
Effects of ZMLpp and TMLpp on the structure and composition of the intestinal microbiota in DSS-treated mice. (**A**,**B**) Boxplots of α-diversity. (**C**) PCA analysis of β-diversity. (**D**) Bar chart of relative abundance at the phylum level. (**E**) Heatmap of relative abundance at the genus level; (**F**–**P**) Relative abundances of *Akkermansia*, *Phascolarctobacterium*, *Lachnoclostridium*, *Roseburia*, *Holdemania*, *Intestinimonas*, *Parasutterella*, *Brachyspira*, and *Veillonella*. The same letters in the figures indicate that there is no significant difference between the data points. Different letters in the figures indicate significant differences (*p* < 0.05) between the data points. Mixed letters in the figures indicate no significant difference with either of the data points. Con: Control; DSS: dextran sodium sulfate; ZMLpp: *Zophobas morio* larvae protein powder; TMLpp: *Tenebrio molitor* larvae protein powder; PCoA: Principal Coordinates Analysis.

The effects of ZMLpp and TMLpp on the α-diversity of the fecal microbiota in DSS-induced colitis mice are shown in Figure 4A,B. The Shannon and Simpson indices were higher in the DSS group than in the Con group. ZMLpp and TMLpp reduced these indices in DSS mice, suggesting that these insect proteins effectively suppressed the overgrowth of conditional pathogens and promoted the recovery of beneficial bacteria. Consistent with the α-diversity results, the β-diversity analysis (Figure 4C) revealed that, compared with the DSS group, the ZMLpp and TMLpp groups were closer to the Con group. At the phylum level (Figure 4D), ZMLpp and TMLpp treatment significantly shifted the phylum composition towards that of the Con group. Changes in intestinal microbiota at the genus level are shown in Figure 4E. Specifically, as shown in Figure 4E–M, ZMLpp and TMLpp increased the relative abundance of beneficial genera, thereby reshaping the intestinal microbiota and alleviating colitis. Regarding conditional pathogens, DSS induction significantly increased the relative abundances of *Parasutterella*, *Brachyspira*, and *Veillonella* in the mouse intestine (Figure 4N–P). In contrast, intervention with ZMLpp and TMLpp significantly reduced the relative abundances of these conditionally harmful bacteria.

**Figure 5:**
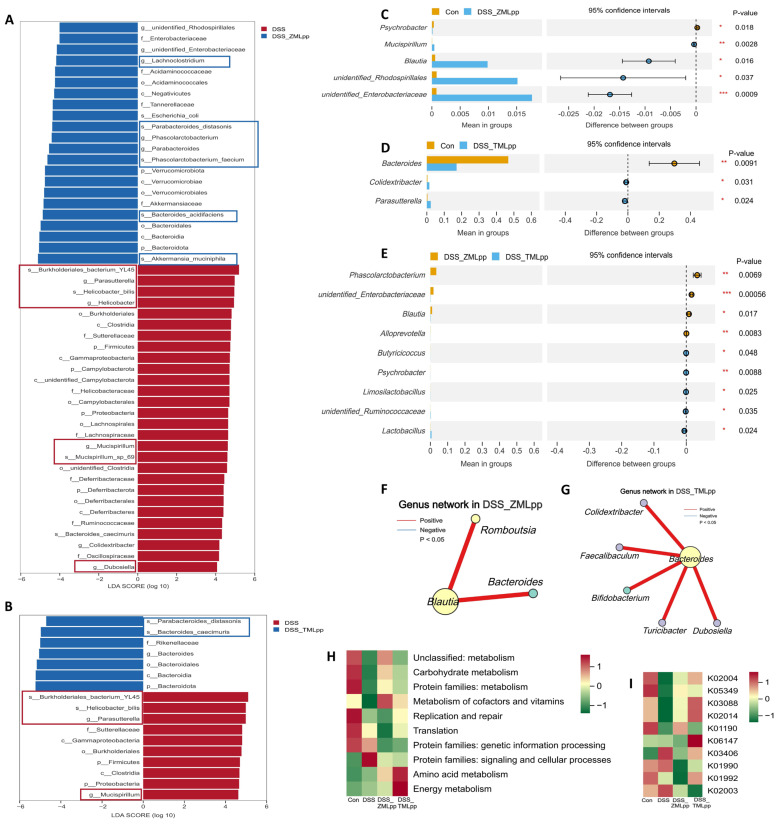
Differential analysis of the intestinal microbiota composition in DSS-treated mice with ZMLpp and TMLpp intervention. (**A**,**B**) LEfSe analysis between the DSS group and the ZMLpp or TMLpp groups, respectively. Microorganisms with an LDA score greater than 4 are displayed as biomarkers significantly contributing to the group separation. (**C**–**E**) *T*-test analysis between the Con group and the ZMLpp/TMLpp groups, and between the ZMLpp and TMLpp groups, respectively (*p* < 0.05). (**F**,**G**) Network Graph of Correlation analysis among microorganisms at the genus level within the ZMLpp and TMLpp groups (*p* < 0.05). (**H**,**I**) Heatmap of functional prediction analysis of microbial KEGG metabolic pathways at level 2 and KO metabolic pathways using PICRUSt2. Con: Control; DSS: dextran sodium sulfate; ZMLpp: *Zophobas morio* larvae protein powder; TMLpp: *Tenebrio molitor* larvae protein powder; LEfSe: Linear discriminant analysis Effect Size; LDA: Linear discriminant analysis; PICRUSt2: Phylogenetic investigation of communities by reconstruction of unobserved states. * *p* < 0.05, ** *p* < 0.01, *** *p* < 0.001.

LEfSe analysis was used to compare the microbiota of the DSS group with that of the ZMLpp and TMLpp intervention groups separately. As shown in Figure 5A, various conditional pathogens were identified as biomarkers for the DSS group. In contrast, various beneficial bacteria were identified as biomarkers for the ZMLpp group. As shown in Figure 5B, various conditional pathogens were identified as biomarkers for the DSS group, while various beneficial bacteria were identified as biomarkers for the TMLpp group. The detection indicates that DSS induction increased inflammatory markers in mice, disrupted intestinal barrier function, increased intestinal permeability, and, consequently, led to a state of microbial imbalance.

T-test analysis comparing the microbiota of the Con group mice and ZMLpp-intervened mice revealed that the ZMLpp group had significantly higher abundances of *Mucispirillum*, *Blautia*, *unidentified_Rhodospirillales*, and *unidentified_Enterobacteriaceae* (Figure 5C). *T*-test analysis comparing the microbiota of the Con group mice and TMLpp-intervened mice revealed that the TMLpp group had significantly higher abundances of *Colidextribacter* and *Parasutterella* (Figure 5D). Although *Mucispirillum*, associated with inflammation, remained significantly higher in the ZMLpp group than in the Con group, *Blautia*, associated with acetate production, was significantly higher and had a greater relative abundance than *Mucispirillum*. This demonstrates the effective reparative effect of ZMLpp on DSS-induced dysbiosis. Pearson correlation analysis supports this view (Figure 5F), showing significant positive correlations between *Blautia* and both *Romboutsia* and *Bacteroides*. *Parasutterella* is associated with DSS-induced inflammatory damage. TMLpp inhibited the abundance of *Parasutterella*, but residual levels were higher than in the Con group. This suggests that the repair mode of TMLpp for DSS damage (strengthening Bacteroidota function) might be slightly less effective than the comprehensive ecological remodeling of ZMLpp. Pearson correlation analysis supports this view (Figure 5G). Functional prediction of Kyoto Encyclopedia of Genes and Genomes (KEGG) and Gene Ontology (GO) metabolic pathways of the microbiota was performed using Phylogenetic investigation of communities by reconstruction of unobserved states (PICRUSt2) (Figure 5H,I) to further analyze the potential impact of ZMLpp and TMLpp’s microbial regulation on host metabolic functions. Compared with the DSS group, the addition of ZMLpp upregulated carbohydrate metabolism, cofactor and vitamin metabolism, and amino acid metabolism. This is related to ZMLpp’s enrichment of short-chain fatty acid (SCFA)-producing bacteria. TMLpp’s regulation of microbial structure was primarily reflected in the upregulation of amino acid metabolism and energy metabolism, providing the foundation for energy-intensive barrier repair and immune cell activity.

## 3. Discussion

Against the backdrop of the growing global interest in novel edible resources, edible insects have emerged as a prominent research focus. Edible insects were found to be highly nutritious and to represent good sources of protein, fat, minerals, vitamins, and energy; they possess advantages such as a short growth cycle, strong reproductive capacity, and low production costs [27]. As a result, they have become a vital source of high-quality nutritional protein for humans, livestock, and poultry. In recent years, studies have revealed that edible insects contain abundant bioactive substances. These substances have been reported to play significant roles in the antioxidant, antihypertensive, antidiabetic, antiobesity, anti-inflammatory, hypocholesterolemia, antimicrobial, anti-severe acute respiratory syndrome coronavirus type 2, antithrombotic, and immunomodulatory properties [17]. These properties endow edible insects with broad application prospects in the medical field.

Currently, drugs used to treat IBD still have notable side effects and limited efficacy. Therefore, the search for safe, highly effective new therapeutic agents remains a core focus of current IBD research. Although there have been reports on some insect extracts that can improve intestinal inflammation [28], studies on whether *Zophobas morio* larvae and *Tenebrio molitor* larvae—as typical edible insects—can alleviate DSS-induced colitis are scarce. This study systematically investigated the alleviating effects of protein powders derived from ZMLpp and TMLpp on DSS-induced colitis in mice, and the findings provide novel insights into the potential of edible insect proteins as safer, natural alternatives for IBD management.

The core manifestations of DSS-induced colitis in mice—including elevated DAI scores, weight loss, hematochezia, diarrhea, and colon shortening—are well-established proxies for human IBD pathogenesis, particularly ulcerative colitis [29,30]. In our study, ZMLpp or TMLpp supplementation by gavage significantly reduced DAI scores, mitigated weight loss, ameliorated gastrointestinal symptoms, and restored colon length, thereby repairing mucosal damage and reducing inflammatory cell infiltration compared to the DSS-only group. These results align with a recent study by Park et al. [26], which demonstrated that *Tenebrio molitor* larvae powder alleviated DSS-induced colitis in mice by improving general disease severity. Notably, our research extends this finding by confirming that *Zophobas morio* larvae protein—previously only reported to have in vitro anti-inflammatory activity [20]—exerts comparable in vivo protective effects against colitis, filling a critical gap in existing knowledge about the therapeutic potential of this understudied edible insect species.

Histopathological analysis further validated the protective role of ZMLpp and TMLpp in preserving intestinal mucosal integrity. The DSS group exhibited severe epithelial destruction, crypt loss, goblet cell depletion, and extensive inflammatory cell infiltration—pathological features consistent with disrupted intestinal barrier function, a hallmark of IBD [31]. In contrast, ZMLpp and TMLpp treatment reduced mucosal damage, preserved crypt structure, and restored goblet cell numbers. Goblet cells are essential for secreting mucin, which forms the primary physical barrier against luminal pathogens and toxins; their preservation suggests that insect proteins may reinforce the intestinal barrier, a mechanism also observed in studies of other natural anti-colitic agents [32]. Additionally, the trend toward reduced spleen weight in treated groups implies a potential modulatory effect on systemic immune activation, as splenomegaly in DSS colitis reflects excessive immune cell proliferation and trafficking to inflamed tissues [33].

The anti-inflammatory mechanisms of ZMLpp and TMLpp were further elucidated at the cellular and molecular levels. Immunohistochemical staining revealed reduced infiltration of leukocytes (via LCA staining) and neutrophils (via MPO staining) in treated mice. Neutrophils are key mediators of acute intestinal inflammation, and MPO activity is a well-recognized biomarker of neutrophil-mediated tissue damage in IBD [34]. The suppression of these cells by ZMLpp and TMLpp is consistent with their ability to downregulate the mRNA expression of pro-inflammatory genes (*Ccl2*, *Cxcl1*, *Ptgs2*, and *Nf-κb*) via RT-qPCR. *Ccl2* and *Cxcl1* are chemokines that recruit monocytes and neutrophils to inflamed tissues, while *Ptgs2* encodes cyclooxygenase-2 (COX-2), an enzyme critical for synthesizing pro-inflammatory prostaglandins [35]. Most notably, *Nf-κb* is a master transcription factor that regulates the expression of hundreds of pro-inflammatory genes; its inhibition is a central mechanism in effective IBD therapy [36]. These molecular findings parallel the anti-inflammatory properties of *Tenebrio molitor* reported in vitro, where its extracts inhibited NF-κB activation [37], and extend this mechanism to *Zophobas morio* for the first time.

A critical advantage of edible insects as therapeutic agents lies in their nutritional and bioactive composition. They are typically rich in high-quality proteins, unsaturated fatty acids, chitin, and antimicrobial peptides (AMPs)-all of which may contribute to their anti-colitic effects [12,38]. High-quality proteins provide amino acid precursors for mucosal repair, while chitin (a polysaccharide) has been shown to modulate gut microbiota and enhance intestinal barrier function by upregulating tight junction proteins [39]. AMPs, such as cecropins and defensins, exhibit broad antimicrobial and immunomodulatory activities [40,41], which reduce intestinal inflammation by suppressing pro-inflammatory cytokine production. While our study did not isolate specific active components, the retention of anti-colitic activity in ZMLpp and TMLpp—even after defatting and protein purification—suggests that bioactive peptides (rather than lipids or chitin alone) may be key mediators, consistent with [20], who found that *Zophobas morio* protein-derived peptides retained anti-inflammatory activity after extraction.

The potential of ZMLpp and TMLpp to improve gut microbiota dysbiosis—another core driver of IBD [42]—warrants further discussion. Although the results of 16S rRNA sequencing are not fully detailed here, we have observed that DSS-induced colitis leads to severe dysbiosis of the gut microbiota, increasing the chaos within the microbial community. This may be related to DSS disrupting the intestinal mucosal barrier, triggering intense inflammation, and altering the intestinal environment [43]. During the experimental period, both insect protein treatments alleviated this condition, shifting the microbial structure toward a more ordered and healthy state. In terms of mechanisms, both treatments share commonalities while also having distinct emphases: they both reversed the dysbiosis caused by DSS (e.g., reducing the Firmicutes/Bacteroidota ratio and inhibiting Proteobacteria) and increased the abundance of several butyrate-producing genera (such as *Lachnoclostridium* and *Roseburia*). ZMLpp has a more comprehensive repair pathway for DSS-induced bodily damage, functioning through two approaches: promoting the repair of intestinal barrier function and increasing SCFA levels. It is speculated that the effect of enriching SCFA-producing bacteria not only enhances the metabolic function of mice but may also provide the ability to resist the accumulation of inflammatory factors by accumulating SCFAs [44]. In contrast, TMLpp’s repair pathway for DSS-induced bodily damage focuses on enhancing the functions of Bacteroidetes, primarily through the enrichment of two core beneficial strains, *Parabacteroides* distasonis [45] and *Bacteroides* caecimuris, which play a role in maintaining intestinal immune homeostasis. The regulatory effect of TMLpp on the microbial structure is mainly reflected in the upregulation of amino acid metabolism and energy metabolism, providing a foundation for high-energy barrier repair and immune cell activity. These results collectively suggest that ZMLpp and TMLpp alleviate DSS-induced colitis by reshaping the gut microbiota structure and promoting the recovery of beneficial functional bacteria.

This study has several limitations that must be addressed. First, the active components in ZMLpp and TMLpp—such as specific peptides or AMPs—have not been isolated or characterized, precluding definitive identification of their molecular targets. Second, the study was conducted exclusively in mice; translational research in human IBD patients is needed to validate efficacy and safety, considering the numerous differences—genetic/gut microbiota and otherwise—between humans and mice. Moreover, the study lacks a positive control group treated with a standard anti-inflammatory drug (e.g., S-ASA or Sulfasalazine). Therefore, it is difficult to gauge the clinical relevance or the relative efficacy of the insect proteins compared to established therapies. Third, dose–response relationships were not explored; optimizing dosages could enhance therapeutic potential and minimize potential side effects. Finally, the long-term effects of ZMLpp and TMLpp—including their ability to prevent colitis relapse—remain untested, a critical consideration for chronic diseases like IBD.

In conclusion, this study demonstrates that ZMLpp and TMLpp alleviate DSS-induced colitis in mice by reducing disease severity, preserving intestinal mucosa, suppressing inflammatory cell infiltration, downregulating pro-inflammatory gene expression, and regulating fecal microbial structure. These effects likely stem from the bioactive components of insect proteins, such as peptides and AMPs, which act via multiple mechanisms to target IBD pathogenesis. Given the emerging evidence for *Zophobas morio*’s bioactivity and the safety of *Tenebrio molitor* as an edible resource [46], insect proteins represent a promising, sustainable avenue for developing novel functional foods or adjuvant therapies for IBD. Future research should focus on isolating active components, conducting clinical trials, and exploring synergistic effects with existing IBD medications to improve patient outcomes.

## 4. Materials and Methods

### 4.1. Materials

ZML and TML were obtained from Qingdao Xinnongkang Bioengineering Co., Ltd. (Qingdao, China) and Guizhou Liangji Agricultural Technology Co., Ltd. (Guiyang, China), respectively. SPF six-week-old male Kunming mice (35–40 g) were purchased from Chongqing Tengxin Biotechnology Co., Ltd. (Chongqing, China). DSS was purchased from MCE (Monmouth Junction, NJ, USA). The immunohistochemical antibodies LCA antibodies and MPO antibodies were purchased from Proteintech (Wuhan, China).

### 4.2. The Preparation of ZMLpp and TMLpp

ZML and TML were dried and subsequently ground into a fine powder using a grinder. The ground material was then passed through a 60-mesh sieve to collect the insect larval powder. For defatting, petroleum ether was added to the larval powder at a solid-to-liquid ratio of 1:10 (*w*/*v*, g/mL), followed by ultrasound-assisted defatting treatment for 24 h. After defatting, the mixture was vacuum-filtered to separate the solid fraction, which was then air-dried to obtain defatted insect larval powder. Next, proteins were extracted from the defatted powder via an ultrasound-assisted alkaline extraction method. The resulting protein extract was further processed by acid precipitation, repeated washing, and drying to yield defatted protein powder (i.e., ZMLpp or TMLpp), which was stored at 4 °C in a refrigerator until use. For animal administration, a stock solution of the protein powder was prepared by dissolving it in normal saline. The amino acid composition of the protein powder was determined using an amino acid analyzer (Biochrom, Cambridgeshire, UK), and the specific components are detailed in Tables S1 and S2.

### 4.3. DSS-Induced Mice Colitis and Treated with ZMLpp or TMLpp

Thirty-two male mice were placed in a specific pathogen-free (SPF) room maintained at an air-conditioned temperature (23–25 °C) and a relative humidity of 50–60% on a 12 h light/dark cycle. Subsequently, they were randomly divided into four groups (n = 8): Control group (standard water), DSS group (3% DSS in water), DSS_ZMLpp group (3% DSS in water +ZMLpp-treated), and DSS_TMLpp group (3% DSS in water +TMLpp-treated). All mice were fed the maintenance feed. During the experiment, all mice were allowed free access to food and water. After a 7-day acclimation period, mice in the DSS_ZMLpp and DSS_TMLpp groups were orally gavaged with 200 μL of ZMLpp (200 mg/kg per day) or TMLpp (150 mg/kg per day), respectively, for 14 consecutive days. The selection of these doses is based on our preliminary acute toxicity studies of ZMLpp (maximum dose of 4000 mg/kg) and TMLpp (maximum dose of 3000 mg/kg), as well as a subchronic toxicity study. In this subchronic toxicity study, mice were gavaged with 200 mg/kg of ZMLpp (1/20 of the maximum dose) and 150 mg/kg of TMLpp (1/20 of the maximum dose) for 90 days. In both studies, no signs of toxicity were observed in the mice, and the treatments did not affect their food intake or behavior. Meanwhile, mice in the Con and DSS groups were given an equal volume of the corresponding solvent (saline) by intragastric administration daily. Then, 3% DSS in water was given to the mice in the DSS, DSS_ZMLpp, or DSS_TMLpp groups for 7 subsequent days. During this period, the mice in DSS_ZMLpp or DSS_TMLpp groups continued to receive gavage with ZMLpp or TMLpp. The mice were weighed, and their stool, hematochezia, and health status were observed daily. On the 22nd day, all mice were euthanized by CO_2_ asphyxiation. The spleen was quickly excised and weighed. The colon was dissected, its length was measured, and it was cut open longitudinally for further use. The fecal samples were collected, snap-frozen in liquid nitrogen, and stored at −80 °C for later analysis. The experimental treatment procedure is clearly depicted in Figure 1A, drawn by Figdraw (Hangzhou, China).

### 4.4. DAI Score

DAI score were determined based on the scores of rectal bleedings (0, no observed blood; 1, a small amount of blood in stool; 2, blood in stool regularly seen; and 3, blood in all stool), stool consistency (0, regular; 1, soft but still formed; 2, very soft; and 3, diarrhea) and body weight loss (0, no loss; 1, 1–5% loss; 2, 5–10% loss; and 3, 10–20% loss) as described methods [47].

### 4.5. Histopathological Score of Colon Tissue

Colon tissue was fixed in 10% neutral-buffered formalin, embedded, sectioned into 4 μm-thick sections, and attached to slides. Tissue slides were stained with H&E and evaluated for inflammatory cell infiltration, crypt depletion, and the integrity of the epithelial barrier. Periodic acid-Schiff (PAS) staining was performed to confirm a decrease in the number of goblet cells. Histopathological score of colon is based on the epithelium (0, normal; 1, loss of goblet cells; 2, loss of goblet cells in large areas; 3, loss of crypts; and 4, loss of crypts in large areas) and infiltration (1, infiltrate around crypt basis; 2, infiltrate reaching the mucosae; 3, extensive infiltration into the mucosae; and 4, infiltration of the submucosa), as previously studied [48].

### 4.6. Immunohistochemical (IHC) Staining

IHC staining for LCA and MPO was performed to detect and quantify leukocytes and neutrophils in the colon, respectively. Briefly, the sections were treated with blocking buffer (Dako Denmark A/S, Glostrup, Denmark) for 30 min at RT and then incubated overnight at 4 °C with anti-LCA (1:300) or anti-MPO (1:100) antibodies, respectively. After thorough rinsing with Tris-NaCl buffer, the sections were incubated with biotinylated goat anti-rabbit IgG (1:200 dilution in Tris-NaCl buffer) for 60 min at RT. The sections were subsequently incubated with an avidin–biotinylated enzyme complex and 3,3’-diaminobenzidine (DAB). The quantification of leukocytes and neutrophils in the colon of each group was carried out as follows. Under a 200× magnification field of view, eight non-overlapping fields of view were randomly selected in the colon of each mouse. The number of LCA-positive expression cells or MPO-positive expression cells was counted, and the average values were taken.

### 4.7. Real-Time Quantitative PCR (RT-qPCR)

The mRNA levels of inflammatory cytokines *Ccl2*, *Cxcl1*, *Ptgs2*, and the *Nf-κb* gene in colon tissues were detected by RT-qPCR. Total RNA was extracted from the mouse colon with TRIzol reagent (Invitrogen, Carlsbad, CA, USA). The RNA was reverse transcribed to cDNA and then subjected to RT-qPCR. RT-qPCR was performed by using SYBR Green Premix Pro Taq HS qPCR Kit (AG, Changsha, China). Primer sequences are shown in Table 1. The relative mRNA levels were calculated by 2^−ΔΔCt^.

### 4.8. Analysis of Gut Microbial

Mice fecal microbiota DNA was isolated by using the DNeasy PowerSoil Pro kit (Qiagen, Shanghai, China). Samples were then assessed using 16S rRNA sequencing, as in a previous study with modifications. Briefly, the 16S rRNA genes in the V3–V4 region were amplified with barcoded primers (341F and 806R). Amplified PCR products were purified, quantified, and pooled to the same equimolar concentration. Sequencing analysis was performed on the Illumina MiSeq platform (Illumina, Inc., San Diego, CA, USA) after combining the amplicon library.

### 4.9. Statistical Analysis

Data from the groups are expressed as the means ± standard deviations (SD). Means were compared using a one-way analysis of variance (ANOVA) and a *t*-test in SPSS version 20.0 (IBM, Armonk, NY, USA) to determine whether there were statistically significant differences. A *p*-value < 0.05 indicated a statistically significant difference.

## Figures and Tables

**Table 1:** Primer Sequences.

Species	Genes	Forward Primer	Reverse Primer
mice	*Actin beta*	CCTGGCGATACCTCAGCAACC	CCTCCACGGCTCAACCACTG
	*Ccl2*	CCGGCTGGAGCATCCACGTGT	TGGGGTCAGCACAGACCTCTCTCT
	*Cxcl1*	CAATGAGCTGCGCTGTCAGT	TTGAGGTGAATCCCAGCCAT
	*Ptgs2*	TCCAACCTCTCCTACTACACCAG	GGGTCAGGGATGAACTCTCTC
	*Nf-κb*	AGGCTTCTGGGCCTTATGTG	TGCTTCTCTCGCCAGGAATAC

## Data Availability

The original contributions presented in this study are included in the article/Supplementary Material. Further inquiries can be directed to the corresponding author.

## Supplementary Materials

The following supporting information can be downloaded at https://www.mdpi.com/article/10.3390/ijms27031405/s1.

## Abbreviations

The following abbreviations are used in this manuscript:

Inflammatory Bowel Disease (IBD); *Zophobas morio* larvae (ZML); *Tenebrio molitor* larvae (TML); ZML protein powder (ZMLpp); TML protein powder (TMLpp); European Food Safety Authority (EFSA); Dextran sodium sulfate (DSS); Disease Activity Index (DAI); Antimicrobial peptides (AMPs); Hematoxylin and eosin (H&E); Periodic acid-Schiff (PAS); Leukocyte Common Antigen (LCA); Myeloperoxidase (MPO); Kyoto Encyclopedia of Genes and Genomes (KEGG); Gene Ontology (GO); Phylogenetic investigation of communities by reconstruction of unobserved states (PICRUSt2); Principal Coordinates Analysis (PCoA); Linear discriminant analysis Effect Size (LEfSe); Linear discriminant analysis (LDA).
